# iTRAQ-based proteomic analysis reveals key proteins affecting muscle growth and lipid deposition in pigs

**DOI:** 10.1038/srep46717

**Published:** 2017-04-24

**Authors:** Zhixiu Wang, Peng Shang, Qinggang Li, Liyuan Wang, Yangzom Chamba, Bo Zhang, Hao Zhang, Changxin Wu

**Affiliations:** 1 National Engineering Laboratory for Animal Breeding, China Agricultural University, No. 2 Yuanmingyuan Xilu, Beijing 100193, China; 2 College of Animal Science, Tibet Agriculture and Animal Husbandry University, Linzhi, 100086, China; 3 Institute of Animal Sciences and Veterinary Medicine, Anhui Academy of Agricultural Sciences, Hefei 230031, China

## Abstract

Growth rate and meat quality, two economically important traits in pigs, are controlled by multiple genes and biological pathways. In the present study, we performed a proteomic analysis of *longissimus dorsi* muscle from six-month-old pigs from two Chinese native mini-type breeds (TP and DSP) and two introduced western breeds (YY and LL) using isobaric tag for relative and absolute quantification (iTRAQ). In total, 4,815 peptides corresponding to 969 proteins were detected. Comparison of expression patterns between TP-DSP and YY-LL revealed 288 differentially expressed proteins (DEPs), of which 169 were up-regulated and 119 were down-regulated. Functional annotation suggested that 28 DEPs were related to muscle growth and 15 to lipid deposition. Protein interaction network predictions indicated that differences in muscle growth and muscle fibre between TP-DSP and YY-LL groups were regulated by ALDOC, ENO3, PGK1, PGK2, TNNT1, TNNT3, TPM1, TPM2, TPM3, MYL3, MYH4, and TNNC2, whereas differences in lipid deposition ability were regulated by LPL, APOA1, APOC3, ACADM, FABP3, ACADVL, ACAA2, ACAT1, HADH, and PECI. Twelve DEPs were analysed using parallel reaction monitoring to confirm the reliability of the iTRAQ analysis. Our findings provide new insights into key proteins involved in muscle growth and lipid deposition in the pig.

Pigs are a major source of dietary protein and have considerable value for biomedical research^1^,^2^,^3^,^4^. Multiple genes and biological pathways control growth rate and meat quality, which are the most economically important traits. Numerous studies have used high-throughput technologies to analyse the genome, transcriptome, and proteome in order to provide insights into the mechanisms of muscle development in pigs.

Isobaric tag for relative and absolute quantification (iTRAQ) is a powerful technique for quantitative analysis of proteomes. It can identify numerous proteins and quantify them more reliably than the traditional two-dimensional electrophoresis^5^. Further, information on a sufficient number of proteins might allow pathway and protein-protein interaction analyses^6^. Recent studies have shown that iTRAQ can be used to analyse protein abundance in tender and tough meat from the bovine *longissimus thoracis* muscle^7^. Several recent studies have focused on the pig proteome from a range of tissues and cells such as liver^8^,^9^, skeletal muscles^10^, *longissimus dorsi* (LD) from the early embryonic stage^11^, heart^12^, pulmonary alveolar macrophages^13^, intestine^14^, mesenchymal stem cells^15^, and brainstem^16^. For example, 4,431 proteins were identified using iTRAQ-based proteome analysis of early embryonic LD muscle (21 to 42 days post coitus) from Landrace and Wuzhishan breeds^17^.

Parallel reaction monitoring (PRM) is a recent development in targeted mass spectrometry, which involves the use of a quadrupole-equipped orbitrap^18^. This method is more specific and sensitive than selected reaction monitoring and has been widely used to quantify and detect target proteins^19^,^20^,^21^.

The Tibetan pig (TP) and Diannan Small-Ear pig (DSP) are indigenous Chinese breeds; they have significantly lower growth rates, higher lipid deposition ability, and better meat quality than those of introduced pig breeds, such as Yorkshire (YY) and Landrace (LL)^22^,^23^,^24^,^25^. In our previous study, we identified genes related to growth and lipid deposition from the transcriptome profiles of LD muscles of pigs^25^. In the present study, we performed iTRAQ-based quantitative proteome analysis of the Chinese indigenous (TP and DSP) and introduced (YY and LL) pig breeds to identify functional proteins associated with muscle growth and lipid deposition pathways. Additionally, PRM was used to confirm the iTRAQ results. Our findings are likely to enhance our understanding of muscle development mechanisms in pigs and other agricultural animals.

## Results

### Histology and histochemistry of the LD muscle

**Figure 1:**
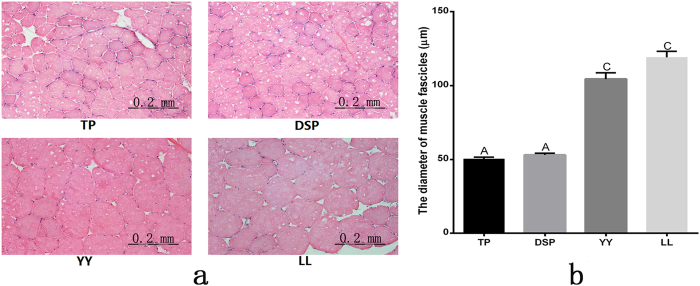
Histological analysis of the muscle tissues. (**a**) HE-stained longitudinal sections of the LD muscle, and (**b**) the diameter (μm) of muscle fascicles in four breeds of pig. The bars indicate the standard error. Different letters indicate significant differences (P < 0.01).

The results of histological and histochemical analysis of the LD muscle in the four pig breeds (TP, DSP, YY, and LL) are shown in Fig. 1(a and b). The diameter of the LD muscle fascicles was significantly smaller in TP and DSP than in YY and LL (P < 0.01).

### Protein identification and quantification

**Figure 2:**
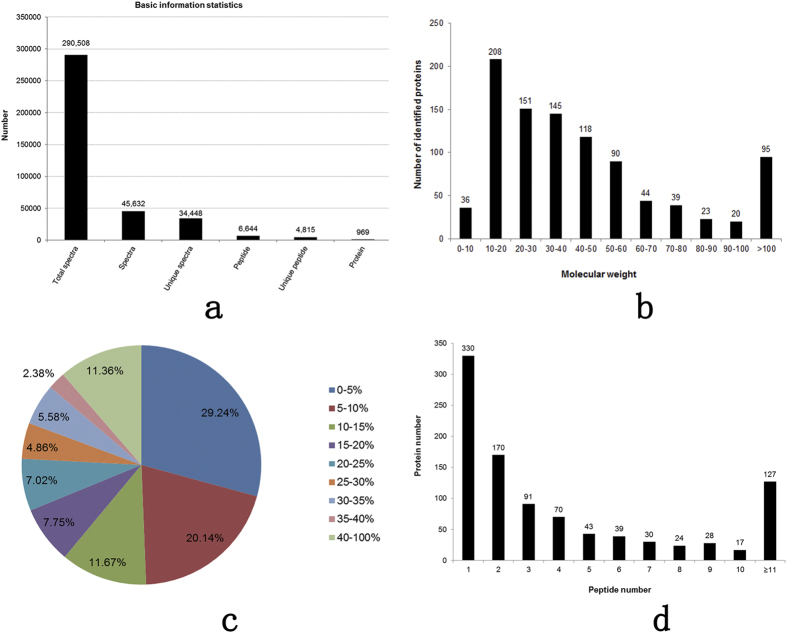
Protein identification and analysis. (**a**) Basic information of protein identification, (**b**) distribution of the identified proteins among the different molecular weight classes (in kD), (**c**) coverage of proteins by the identified peptides, and (**d**) distribution of proteins containing different number of identified peptides.

An 8-plex LC-MS/MS analysis produced 290,508 spectra (Fig. 2a), corresponded to 4,815 unique peptides (Fig. 2a; see Supplementary Table S1), and 969 proteins were identified at a false discovery rate (FDR) of ≤0.01 (Fig. 2a; see Supplementary Table S2). Most of the identified proteins (73.48%) had molecular weights in the range of 10–20 kD (208), 20–30 kD (151), 30–40 kD (145), 40–50 kD (118), or 50–60 kD (90) (Fig. 2b). In addition, the identified proteins had high peptide coverage, of which 51 and 31% showed more than 10 and 20% sequence coverage, respectively (Fig. 2c). About 48.35% of the identified proteins had three or more peptides (Fig. 2d).

**Figure 3:**
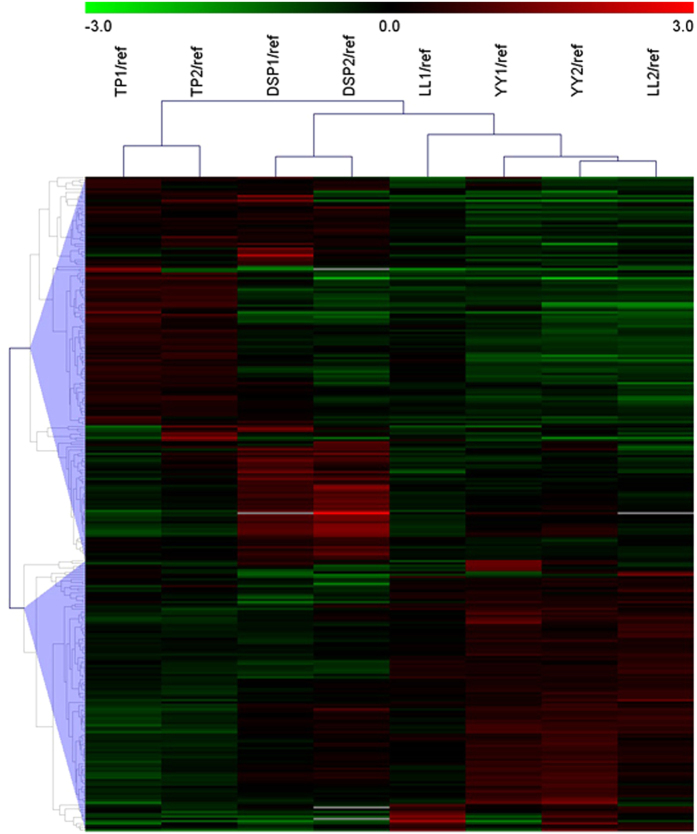
Cluster analysis of DEPs obtained from eight labelled samples.

We identified 288 DEPs, of which 169 were up-regulated and 119 were down-regulated in the TP-DSP group (see Supplementary Table S3). The cluster analysis based on protein abundance data of the 288 DEPs showed that the two biological duplications of each breed cluster into one group and that TP and DSP, and YY and LL cluster together (Fig. 3).

### Functional annotations of the up-regulated DEPs

**Figure 4:**
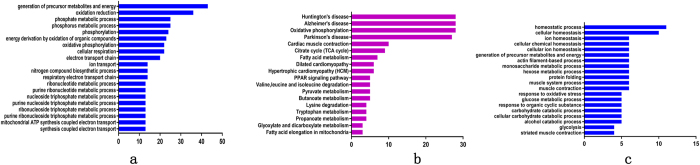
Top terms of functional annotation. (**a**) The top terms of biological processes for the up-regulated DEPs, (**b**) the top terms of KEGG pathway for the up-regulated DEPs, and (**c**) the top terms of biological processes for the down-regulated DEPs.

The 169 up-regulated DEPs in TP-DSP were functionally classified into 35 annotation clusters (see Supplementary Table S4). The top 20 terms of gene ontology (GO) annotation for biological processes showed that most of the up-regulated DEPs participated in precursor metabolite and energy generation, oxidation-reduction, phosphate metabolic process, phosphorylation, energy derivation by oxidation of organic compounds, oxidative phosphorylation, cellular respiration, and electron transport chain (Fig. 4a). The top 20 GO terms for cellular component and molecular function indicated that the up-regulated DEPs were mainly enriched in the mitochondrion, organelle membrane, organelle envelope, inorganic cation transmembrane transporter activity, and cofactor binding, and oxidoreductase activity (see Supplementary Fig. S1). The top Kyoto Encyclopedia of Genes and Genomes (KEGG) pathways of up-regulated DEPs in TP-DSP were involved in oxidative phosphorylation, tricarboxylic acid cycle, fatty acid metabolism, fatty acid elongation in mitochondria, and in peroxisome proliferator-activated receptor (PPAR) signalling pathway (Fig. 4b). Thus, in the TP-DSP group the up-regulated DEPs were mainly enriched in energy and fat metabolism. Furthermore, cluster 9 was related to muscle system process, and clusters 5, 7, 8, 10, 11, 12, and 15 were related to fatty acid metabolism and lipid catabolic process (see Supplementary Table S4). Eight myogenic (Table 1) and 15 lipid-deposition (Table 2) proteins were present among the 169 up-regulated DEPs.

### Functional annotation of down-regulated DEPs

The 119 down-regulated DEPs were functionally classified into 31 annotation clusters (see Supplementary Table S5). The top 20 GO terms for BP showed that most of the down-regulated DEPs were involved in homeostatic process, ion homeostasis, actin filament-based process, muscle system process, muscle contraction, glucose metabolism, and striated muscle contraction (Fig. 4c). The top 20 GO terms for CC and MF showed that the down-regulated DEPs were mainly enriched in non-membrane-bounded organelle, cytoskeleton, actin cytoskeleton, contractile fibre, myofibril, nucleotide binding, ATP binding, and cytoskeletal protein binding (see Supplementary Fig. S2). The KEGG annotation showed that the down-regulated DEPs participated in the glycolysis/gluconeogenesis pathway. In the TP-DSP group, the down-regulated DEPs were mainly enriched in categories related to muscle development. The functional annotation showed that clusters 1, 7, 8, and 9 were associated with actin cytoskeleton, muscle contraction, and skeletal system development, but no obvious fat metabolic clusters were found. Based on these clusters, we identified 20 myogenic DEPs from the 119 down-regulated DEPs (Table 1).

### Protein interaction network for muscle growth and lipid metabolism

**Figure 5:**
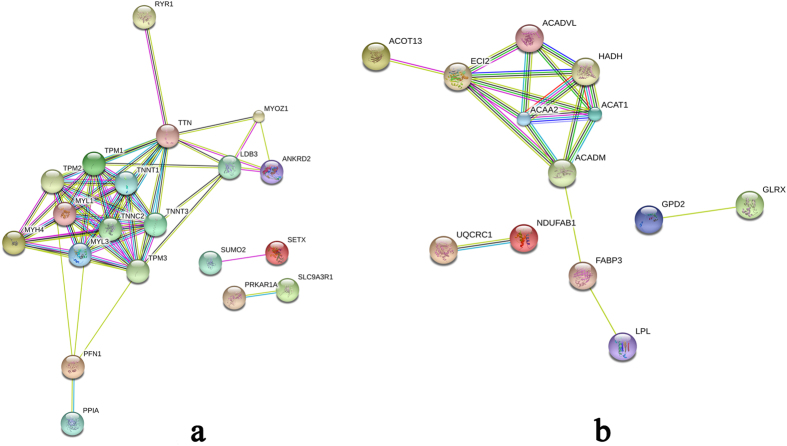
Interaction network of important proteins identified using iTRAQ. (**a**) The interaction network of important myogenic proteins, and (**b**) the interaction network of important lipid deposition proteins. Network nodes represent proteins; small nodes: proteins of unknown three-dimensional structure, large nodes: some three-dimensional structure is known or predicted, coloured nodes: query proteins and first shell of interactors, and white nodes: second shell of interactors. Edges represent protein-protein associations; known interactions: 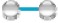 from curated databases, and 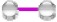 experimentally determined. Predicted interactions: 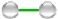 gene neighbourhood; 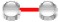 gene fusions; 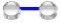 gene co-occurrence. Others: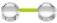 text-mining; 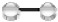 co-expression; 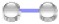 protein homology.

Several strong interactions were found among the DEPs related to muscle growth (Fig. 5a). The TNNT1, TNNT3, TPM1, TPM2, TPM3, MYL3, MYH4, and TNNC2 proteins had pivotal roles in the interaction network. In addition, they might have important roles in regulating muscle growth in pigs. The prediction of the protein interaction network of DEPs related to lipid metabolism showed that ACADVL, ACADM, ACAA2, ACAT1, HADH, and ECI2 had a pivotal role in the network (Fig. 5b). In addition, they might have an important role in regulating lipid metabolism and deposition in pig.

### Validation of DEPs by PRM

The PRM assay was used to confirm the identity several DEPs identified in the iTRAQ analysis. As this assay requires the signature peptide of the target protein to be unique, we only selected proteins with a unique signature peptide sequence for the PRM analysis. Twelve DEPs (up-regulated: UQCRC1, ACAT1, ACADM, PECI, MYL3, NNT, ACAA2, TTN, and HADH; down-regulated: PRDX4, MYL1, and LDB3) were selected for the PRM analysis. The expression values of the up-regulated proteins were higher and those of the down-regulated proteins were lower in the TP-DSP group relative to the YY-LL group (see Supplementary Fig. S3). The fold changes for these proteins were significantly different between the TP-DSP and YY-LL groups at P < 0.10, in agreement with the findings of the iTRAQ analysis (Table 3).

## Discussion

In our previous study, two indigenous Chinese pig breeds (DSP and TP) had significantly lower body weights and higher intramuscular fat (IMF) than those of two introduced pig breeds (LL and YY)^25^. In the present study, we found that the diameter of muscle fascicles was markedly smaller in TP and DSP than in LL and YY, indicating differential muscle development between Chinese and introduced pig breeds. This provides a good model for studying the regulatory mechanisms of muscle development.

Biological processes with particular functions have been identified using proteomic analyses in previous studies of agriculturally important plants and animals^26^,^27^,^28^. In the present study, the proteomic profile of the LD muscle of two indigenous Chinese pig breeds and two pig breeds introduced into China were evaluated. Our results provide new insights into muscle development in pig and improve our understanding of the molecular mechanisms associated with growth and lipid deposition.

Muscle development is closely associated with many critical cellular functions and biological processes. Previous studies have shown that many myofibril proteins such as myosin, troponin, and tropomyosin exist as multiple isoforms, and some of them are differentially expressed in various types of muscle fibre^29^. About one-third of the total muscle proteins are made up of myosin, which is the most abundant protein in muscles^30^,^31^. Different myosins are involved in the differentiation of muscle fibres; type I (slow-twitch, red muscle, oxidative), type IIa (fast-twitch, red muscle, oxidative), and type IIb/IIx (fast-twitch, white muscle, glycolytic). Myosin heavy chain 4 (MYH4 or MyHC IIb) fibres are the most prominent in pigs and contribute to differential growth of muscles^32^. Furthermore, mRNA abundance of MyHC IIb can be used as an indicator of muscle protein synthesis and muscle growth rate^33^,^34^. MyHC IIb is a type of fast fibre, and some studies have shown that the proportion of IIb glycolytic fibres is about 75–86% in commercial pig breeds that have a rapid growth rate^35^. Myosin, light polypeptide 1 (*MYL1*) encodes a myosin alkali light chain, which is expressed in the fast skeletal muscle, and myosin light chains 3 (*MYL3*) encodes an alkali light chain, referred to as the slow skeletal muscle isoform. Previous studies have shown that skeletal muscles of Meishan pigs (another indigenous Chinese pig breed) contain high proportions of slow fibres at slaughter^36^, which are thought to have an important role in determining meat quality^37^. In the present study, MYH4 and MYL1 were down-regulated, and MYL3 was up-regulated in the TP-DSP group. Further, the tropomyosin proteins, tropomyosin alpha-1 chain (TPM1), beta-tropomyosin (TPM2), and tropomyosin 3 (TPM3) were up-regulated, and the troponin proteins, troponin C (TNNC2, fast skeletal muscle type), troponin T (TNNT1, slow skeletal muscle type), and troponin T (TNNT3, fast skeletal muscle type) were down-regulated in the TP-DSP group. The interaction network of muscle growth-related proteins (Fig. 5a) showed that myosin, troponin, and tropomyosin were highly interrelated and played key roles in the entire network. The functional annotation results showed that these proteins were associated with GO terms for myofibril, contractile fibre part, and actin cytoskeleton structures, consistent with the findings from previous research^38^,^39^,^40^.

The down-regulated DEPs were mainly associated with muscle system processes such as muscle contraction, actin cytoskeleton, and glycolysis/gluconeogenesis KEGG pathways. ALDOC, ENO3, PGK1, and PGK2 were involved in the glycolysis/gluconeogenesis signalling pathway. Glycolysis is the process in which glucose is converted to pyruvate, generating small amounts of ATP (energy) and NADH (reducing power), and gluconeogenesis synthesises glucose from non-carbohydrate precursors, implying that this pathway is related to energy metabolism and muscle growth performance^39^. These results showed that these genes contribute to the smaller diameter of LD muscle fibres (Fig. 1) and lower growth rate of the TP-DSP group.

The GO and KEGG enrichment analyses indicated that most of the up-regulated DEPs were associated with fatty acid and lipid metabolism, PPAR signalling pathway, and fatty acid elongation in the mitochondria. The lipoprotein lipase (LPL), apolipoprotein A1 (APOA1), apolipoprotein C3 (APOC3), acyl-CoA dehydrogenase, C-4 to C-12 straight chain (ACADM or MCAD), and fatty acid binding protein 3 (HFABP or FABP3) proteins are known to participate in the PPAR signalling pathway. The LPL protein plays an important role in the metabolism and transport of lipids. It is responsible for the hydrolysis of the triglyceride component in circulating chylomicrons and very low-density lipoprotein (VLDL) by binding to apolipoprotein C2^41^. Several studies have confirmed the association of the *LPL* gene with hypertension^42^, obesity^43^, and insulin resistance^44^. The overexpression of *LPL* in the skeletal muscles leads to excessive intramyocytic lipid deposition, indicating that a relationship exists between lipid storage and insulin sensitivity^45^. In our study, LPL expression in TP-DSP was 2.84-fold higher than that in the YY-LL group, suggesting that it is related to the higher lipid deposition ability of TP and DSP pig breeds. APOA1 and APOC3 are apolipoprotein family members. APOA1 is the major protein component of high-density lipoprotein in the plasma membrane, and APOC3 is a VLDL. A previous study found a significant association between *APOA5* and *APOC3* gene polymorphisms and meat quality traits in Kele pigs^46^.

*ACADM* encodes the medium-chain specific acyl-coenzyme A dehydrogenase. This homotetrameric enzyme catalyses the initial steps of the mitochondrial fatty acid beta-oxidation pathway. A previous study showed that the intragenic synonymous polymorphic variant c.1161A > G in *ACADM* exon 11 (rs1061337) is a functional single nucleotide polymorphism, which causes higher expression of ACADM that might affect fatty acid oxidation^47^. Furthermore, deficiency of MCAD is associated with a severe genetic metabolic disorder that prevents the utilisation of fatty acids^48^. HFABP is thought to participate in the uptake, intracellular metabolism, and/or transport of long-chain fatty acids, and has been associated with, the palatability of pork^49^, and the IMF content and back fat thickness of pigs^50^. The expression of the *HFABP* gene enhances adipogenesis in 3T3-L1 preadipocytes, primarily by up-regulating lipogenic *PPARγ, 422/aP2*, and *GPDH* genes. Recent studies have shown that HFABP is related to adipogenesis in 3T3-L1 preadipocytes of the fat Banna mini-pig inbred line^51^. The distribution of *HFABP* and *ACSL4* gene polymorphisms has been associated with IMF content and back fat thickness in different pig populations^52^. The expression of HFABP in TP-DSP was 1.78-fold higher than that in YY-LL, suggesting that it is related to the higher lipid deposition ability of TP and DSP pig breeds.

Higher expression levels of PPAR signalling pathway related proteins indicate a higher level of lipid metabolism. This result is consistent with those of recent reports^53^. Further, these results were consistent with lower growth rate, enhanced lipid deposition, and better meat quality in TP and DSP than in YY and LL. The network interactions of genes encoding proteins for lipid deposition confirmed a close relationship between ACADVL, ACADM, ACAA2, ACAT1, HADH, and PECI. These proteins participated in the fatty acid metabolism, which is associated with insulin resistance, obesity, and weight loss^54^. These results showed that the combination of these genes was responsible for the high lipid deposition capability of the TP-DSP group.

## Conclusions

In conclusion, we obtained 288 DEPs, of which 28 and 15 were related to muscle growth and lipid deposition, respectively. ALDOC, ENO3, PGK1, PGK2, TNNT1, TNNT3, TPM1, TPM2, TPM3, MYL3, MYH4, and TNNC2 might be involved in the regulation of muscle growth and muscle fibre. LPL, APOA1, APOC3, ACADM, FABP3, ACADVL, ACAA2, ACAT1, HADH, and PECI might affect lipid deposition. Our expression profiles provide new insights into the key proteins involved in muscle growth and lipid deposition in the pig.

## Methods

### Ethics statement

All experiments were conducted according to the guidelines of the China Council on Animal Care, and the protocols used were approved by the animal welfare committee of the State Key Laboratory for Agro-biotechnology of the China Agricultural University (Approval number, XK257).

### Animals and samples

In the present study, the TP and DSP breeds, which are characterised by slow growth and high lipid deposition, were treated as the Chinese pig group (TP-DSP), and the YY and LL breeds, which show rapid growth and lean-mass, served as the introduced pig group (YY-LL). The pigs used were raised in the Beijing Shunyi Pig Breeding Farm. Six individuals from each breed were humanely slaughtered at the age of six months. The LD muscle tissues from the twelfth rib were collected and snap-frozen in liquid nitrogen for the extraction of total proteins.

### Histology and histochemistry of the LD muscle

LD muscles fixed in 4% paraformaldehyde or fresh LD muscles frozen in isopentane cooled by liquid nitrogen were cut into 10-μm thick sections using a cryosectioning machine (CM1900; Leica), and stained with haematoxylin and eosin for morphological analysis. Three images per section and four sections from each pig were analysed. Micrographs were obtained using a digital camera system (CX41; Olympus) and analysed using Image Pro Plus software.

### iTRAQ analysis

Protein was extracted from LD muscle samples, as previously described by Newcom *et al*.^55^. Each frozen sample was ground in liquid nitrogen and suspended in lysis buffer consisting of 8 M urea (U5378; Sigma), 100 mM Tris-HCl (pH 8.0), 10 mM dithiothreitol (DTT), and proteinase inhibitors (4693116001; Roche). The digest was centrifuged at 10,000 × *g* for 30 min at 4 °C, and the supernatant was collected. The concentration of the supernatant was measured, and the protein samples from two pigs of each breed were pooled (1:1) as a biological sample. Next, 200 μg protein was added to 5 μl of 1 M DTT at 37 °C for 1 h and alkylated with 20 μl of 1 M indole acetic acid at room temperature for 1 h in the dark. Trypsin digestion (protein:trypsin ratio of 50:1) was performed for more than 12 h at 37 °C.

The samples were labelled according to the instructions of iTRAQ Reagent-8plex Multiplex Kit (AB SCIEX). Protein samples were labelled as 113 (TP1), 114 (TP2), 115 (DSP1), 116 (DSP2), 117 (YY1), 118 (YY2), 119 (LL1), and 121 (LL2) and then pooled and dried by centrifugal evaporation. The peptides were further fractionated using AKTA Purifier 100 (GE Healthcare) equipped with a strong cation exchange column (4.6 × 100 mm, 5 μm, 200 Å, Polysulfoethyl column; PolyLCInc, Maryland, USA). The retained peptides were eluted with buffer A (10 mM KH_2_PO_4_ in 25% acetonitrile (ACN), pH 3.0) and buffer B (10 mM KH_2_PO_4_, 500 mM KCl, in 25% ACN, pH 3.0) at a flow rate of 1,000 μl/min. Thirty-six fractions were collected and combined to make 10 fractions and reduce peptide complexity, according to protein properties.

### LC-MS/MS analysis

The eluted fractions were lyophilised in a centrifugal speed vacuum concentrator and dissolved with 0.1% formic acid before reversed-phase nanoflow liquid chromatography (nLC) tandem mass spectrometry (nLC-MS/MS). The MS/MS analysis was performed on a high-performance liquid chromatography system. EASY Nano-LC was connected to a hybrid quadrupole/time of-flight mass spectrometer equipped with a nano-electrospray ion source. Peptides from each fraction were equalised to ensure that the same amount of protein from each fraction was mixed and injected into the Nano-LC system. Peptides were separated on a C18 analytical reverse phase column using mixtures of Solution A (0.1% formic acid in water) and Solution B (0.1% formic acid in ACN). The samples were passed through a Thermo Scientific EASY column (2 cm × 100 μm; 5 μm; C18) and separated at a flow rate of 250 nl/min using a Thermo Scientific EASY column (75 μm × 100 mm; 3 μm; C18). Peptide separation was performed using a gradient consisting of 0–80 min from 0% to 40% B, 80–88 min from 40% to 100% B, and 88–100 min of 100% B. A complete MS scan (300–1800 m/z) was acquired in the positive ion mode at a resolution of 70,000 (at 200 m/z), an automatic gain control target value of 3 × 10^6^, a maximum ion accumulation time of 10 ms, number of scan ranges of one, and dynamic exclusion of 40.0 s. Information for peptides and peptide fragments were collected as follows: 10 fragment files collected after every full scan (MS2 scan), higher collision energy dissociation fragmentation, an isolation window of 2 m/z, full scan at a resolution of 17,500 (at 200 m/z), micro-scans of one, maximum ion accumulation time of 60 ms, normalised collision energy of 30 eV, and an under-fill ratio of 0.1%.

### Database search, protein identification, and quantification

The MS/MS data were searched against the uniprot_Sus_scrofa_35257_20151120.fasta database for peptide identification and quantification using Mascot 2.1 and Proteome Discoverer1.4 (Thermo). Proteins with at least two unique peptides and FDR of <0.01 was qualified for further quantification data analysis. The parameters were set as follows: peptide mass tolerance of ±15 ppm, fragment mass tolerance of 20 mmu, number of allowed maximum missed tryptic cleavage sites of two, carbamidomethyl (C) as fixed modification, iTRAQ-8Plex on N-terminal residue, lysine (K), and tyrosine (Y); and acetyl and oxidation on methionine (M) as the variable modification. Proteins were quantified based on the total intensity of the assigned peptides. The average of eight labelled sample mixtures was used as reference (ref), based on the weighted average of the intensity of reported ions in each identified peptide. The final ratios of proteins were normalised according to the median average protein ratio for the mixtures of different labelled samples (TP1/ref, TP2/ref, DSP1/ref, DSP2/ref, YY1/ref, YY2/ref, LL1/ref, and LL2/ref).

We compared the expression levels of all identified proteins between the TP-DSP and the YY-LL groups to identify the proteins involved in muscle growth and lipid deposition. Student’s t-test was used to compare differences of protein expression between the TP-DSP and the YY-LL groups and calculate P values. Fold change of ≥1.3 or ≤0.77 was set as the threshold to identify differently expressed proteins.

### Bioinformatics and statistical analysis

KOBAS 2.0 (http://kobas.cbi.pku.edu.cn/)^56^ and DAVID 6.7 (https://david.ncifcrf.gov/)^57^,^58^ online software were used to perform the GO annotation and KEGG pathway analysis of DEPs between the indigenous and introduced pig groups. The cluster enrichment of GO terms and KEGG pathways was analyzed in the DAVID database. In addition, the function of cluster was judged based on the descriptions of the GO terms and pathways within the cluster. The DEPs related to muscle growth were used to predict protein interactions and construct network by using STRING software (http://string-db.org/).

### PRM-MS analysis

The protein expression levels obtained using iTRAQ analysis were confirmed by quantifying the expression levels of twelve selected proteins by a PRM-MS analysis carried out at the Beijing Bangfei Bioscience Co., Ltd. (Beijing, China). Signature peptides for the target proteins were defined according to the iTRAQ data, and only unique peptide sequences were selected for the PRM analysis. The proteins (60 μg) were prepared, reduced, alkylated, and digested with trypsin following the protocol for iTRAQ analysis. The obtained peptide mixtures were introduced into the mass spectrometer via a C18 trap column (0.10 × 20 mm; 3 μm), and then via a C18 column (0.15 × 120 mm; 1.9 μm).

The MS measurement was performed using a quadrupole mass filter-equipped bench-top Orbitrap mass spectrometer (Q-Exactive; Thermo Scientific). The raw data obtained were then analysed using Proteome Discoverer 1.4 (Thermo Fisher Scientific). The FDR was set to 0.01 for proteins and peptides. Skyline 2.6 software was used for quantitative data processing and proteomic analysis. Four biological replicates were included in each breed in the PRM-MS analysis.

### Statistical analysis

Statistical analysis was performed using SPSS Statistics 22.0. Differences in diameter of LD muscle fascicle among the four breeds were analysed using one-way ANOVA. P < 0.01 was considered extremely significant. Differences analysis in protein expression in the PRM-MS between the TP-DSP and the YY-LL groups were performed using a T-test, and P < 0.10 was taken to indicate statistical significance.

## Additional Information

**How to cite this article:** Wang, Z. *et al*. iTRAQ-based proteomic analysis reveals key proteins affecting muscle growth and lipid deposition in pig. *Sci. Rep.* **7**, 46717; doi: 10.1038/srep46717 (2017).

**Publisher's note:** Springer Nature remains neutral with regard to jurisdictional claims in published maps and institutional affiliations.

## Supplementary Material

Supplementary Information

Supplementary Table S1

Supplementary Table S2

Supplementary Table S3

Supplementary Table S4

Supplementary Table S5

## Figures and Tables

**Table 1:** Differentially expressed proteins (DEPs) related to muscle growth traits in pig.

Gene Symbol	Accession	Description	Fold change	P-value
ANKRD2	G9B792	Ankyrin repeat domain-containing protein 2	1.65	0.06
MYL3	F1SNW4	MYL3	1.58	0.26
TPM1	F2Z5B6	Tropomyosin alpha-1 chain	1.50	0.25
TPM2	Q8MKF3	Beta-tropomyosin (Fragment)	1.50	0.23
PRKAR1A	F1RV23	cAMP-dependent protein kinase type I-alpha regulatory subunit	1.46	0.01
TTN	Q9N251	Titin (Fragment)	1.41	0.08
TPM3	A0A0B8RZ16	Tropomyosin 3	1.40	0.04
HINT1	F1RKI3	Uncharacterized protein	1.30	0.02
LDB3	F1SEN8	Uncharacterized protein	0.77	0.00
NOL8	F1SUF5	Uncharacterized protein	0.77	0.01
PDLIM3	Q6QGC0	PDZ and LIM domain protein 3	0.76	0.00
MYOZ1	Q4PS85	Myozenin-1	0.75	0.03
PFN1	F1RFY1	Profilin	0.75	0.01
PRDX4	F1SQ01	Uncharacterized protein	0.74	0.01
TNNC2	P02587	Troponin C, skeletal muscle	0.72	0.06
SUMO2	P61958	Small ubiquitin-related modifier 2	0.71	0.01
PBXIP1	F1RGP8	Uncharacterized protein (Fragment)	0.70	0.00
SLC9A3R1	B8XH67	Na(+)/H(+) exchange regulatory cofactor NHE-RF	0.70	0.00
MYH4	Q9TV62	Myosin-4	0.66	0.27
FAM3D	I3LS93	Uncharacterized protein	0.64	0.01
PPIA	P62936	Peptidyl-prolyl cis-trans isomerase A	0.63	0.02
TNNT1	Q75ZZ6	Troponin T, slow skeletal muscle	0.63	0.15
MYL1	A1XQT6	MLC1f	0.61	0.07
SETX	F1S0U6	Uncharacterized protein	0.61	0.01
COL9A1	F1RTT3	Uncharacterized protein	0.60	0.00
TNNT3	Q75NH0	Troponin T fast skeletal muscle type	0.54	0.05
MRPS18A	F1RRH6	Uncharacterized protein	0.52	0.00
RYR1	Q6LAA3	Calcium release channel (Ryanodine receptor 1) (Fragment)	0.42	0.07

**Table 2:** DEPs related to lipid deposition traits in pig.

Gene Symbol	Accession	Description	Fold change	P-value
LPL	A0A0B8RZE8	Lipoprotein lipase	2.84	0.10
NDUFAB1	D0G781	Acyl carrier protein	1.85	0.00
H-FABP	H6UI30	Heart fatty acid-binding protein	1.78	0.00
LMNB1	F1RKM0	Uncharacterized protein	1.69	0.07
ACAA2	D0G0B3	Acetyl-coenzyme A acyltransferase 2	1.68	0.13
ACADM	P41367	Medium-chain specific acyl-CoA dehydrogenase, mitochondrial	1.41	0.01
ACOT13	F1RUE0	Uncharacterized protein	1.41	0.00
UQCRC1	F1SKM0	Uncharacterized protein	1.40	0.00
GPD2	M3TYQ8	Glycerol-3-phosphate dehydrogenase	1.39	0.03
GLRX	P12309	Glutaredoxin-1	1.38	0.01
NNT	K9IW80	Nicotinamide nucleotide transhydrogenase	1.37	0.00
HADH	P00348	Hydroxyacyl-coenzyme A dehydrogenase, mitochondrial	1.37	0.12
ACADVL	A0A0B8RTA8	Acyl-CoA dehydrogenase, very long chain	1.35	0.01
ACAT1	I3LP02	Uncharacterized protein	1.34	0.00
PECI	A9X3T3	Peroxisomal D3, D2-enoyl-CoA isomerase	1.32	0.02

**Table 3:** Confirmation of DEPs detected in iTRAQ analysis using PRM analysis.

Accession	Gene Symbol	Fold change (TP-DSP/YY-LL) in iTRAQ	P-value in iTRAQ	Fold change (TP-DSP/YY-LL) in PRM	P-value in PRM
F1SKM0	UQCRC1	1.40	0.0006	3.26	0.0008
I3LP02	ACAT1	1.34	0.0025	3.60	0.0005
P41367	ACADM	1.41	0.0127	3.80	0.0091
A9X3T3	PECI	1.32	0.0194	4.23	0.0069
F1SNW4	MYL3	1.58	0.2596	3.41	0.0042
K9IW80	NNT	1.37	0.0036	3.95	0.0004
D0G0B3	ACAA2	1.68	0.1300	5.00	0.0053
Q9N251	TTN	1.41	0.0751	2.03	0.0521
P00348	HADH	1.37	0.1196	2.57	0.0134
F1SQ01	PRDX4	0.74	0.0074	0.69	0.0523
A1XQT6	MYL1	0.61	0.0686	0.74	0.0527
F1SEN8	LDB3	0.77	0.0010	0.80	0.0647
