# Supplementary material for: iTRAQ-based proteomic analysis reveals key proteins affecting muscle growth and lipid deposition in pigs
Source: Sci Rep. 2017 Apr 24;7:46717. doi: 10.1038/srep46717 (PMC5402282; doi:10.1038/srep46717)
Supplement: Supplementary Information [file srep46717-s1.pdf]

Supplementary Information

iTRAQ-based proteomic analysis reveals key proteins affecting muscle growth and lipid deposition in pig

Zhixiu Wang[1+], Peng Shang[1+, 2], Qinggang Li[3], Liyuan Wang[1], Yangzom Chamba[2], Bo Zhang[1], Hao Zhang[1]*, Changxin Wu[1]

1 *National Engineering Laboratory for Animal Breeding, China Agricultural University, No. 2 Yuanmingyuan West Road, Beijing 100193, China*

2 *College of Animal Science, Tibet Agriculture and Animal Husbandry University, Linzhi, 860000, China*

3 *Institute of Animal Sciences and Veterinary Medicine, Anhui Academy of Agricultural Sciences, Hefei 230031, China*

* Corresponding author

Email: zhanghao827@163.com (HZ)

[+] These authors contributed equally to this work.

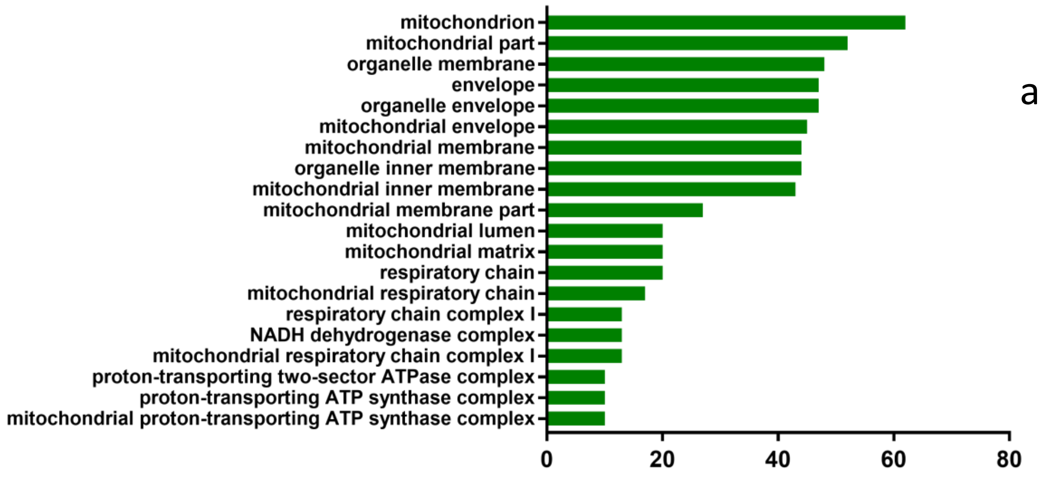

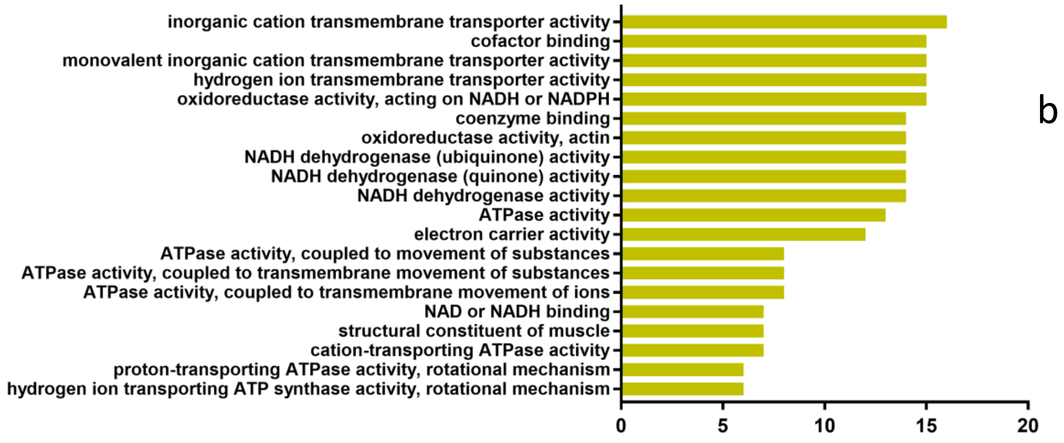

Supplementary figure S1. Top terms of cellular component and molecular function of up-regulated DEPs. Panel (a) shows the top terms of cellular component. Panel (b) shows the top terms of molecular function.

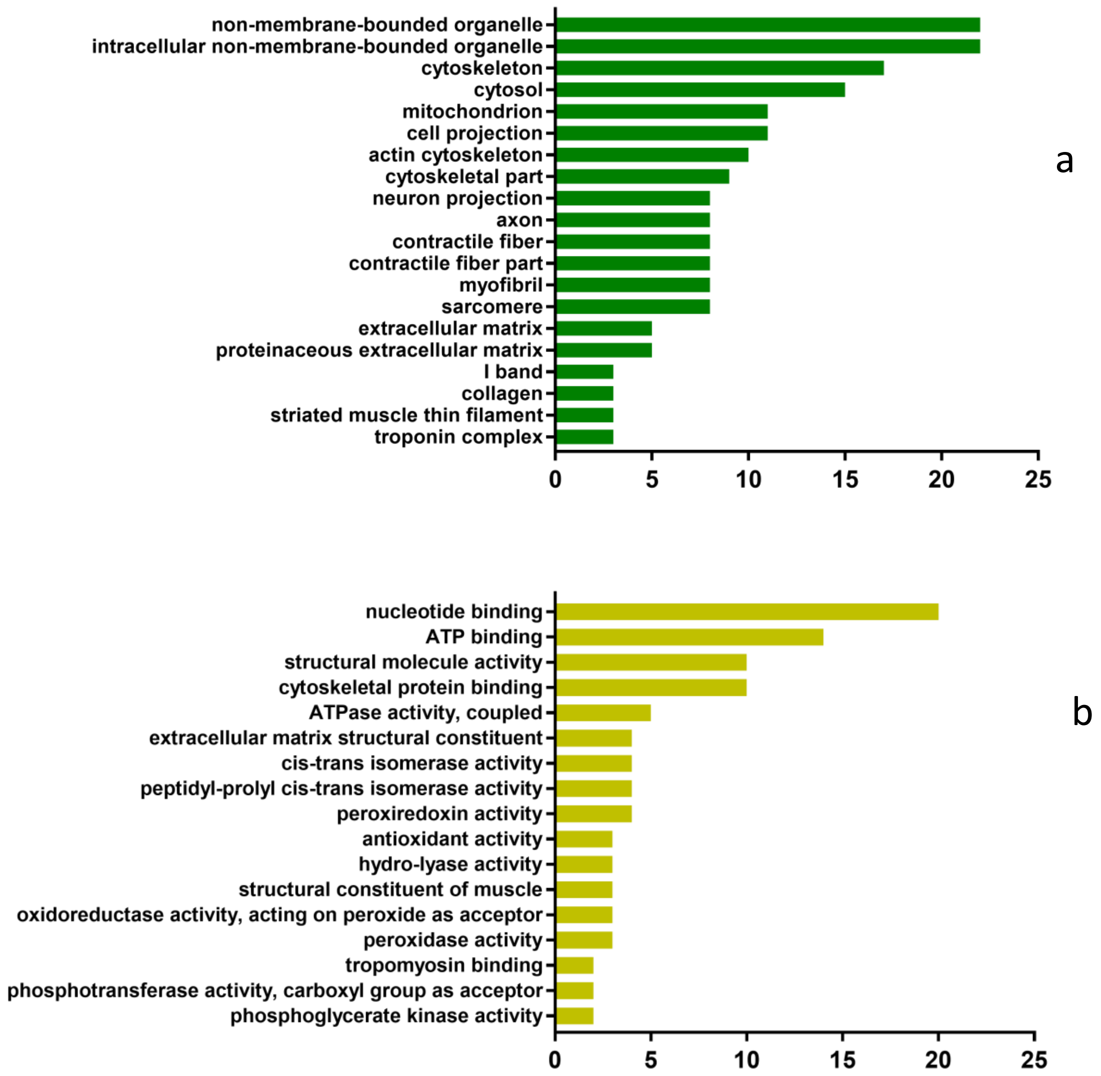

Figure S2. Top terms of cellular component and molecular function of down-regulated DEPs. Panel (a) shows the top terms of cellular component. Panel (b) shows the top terms of molecular function.

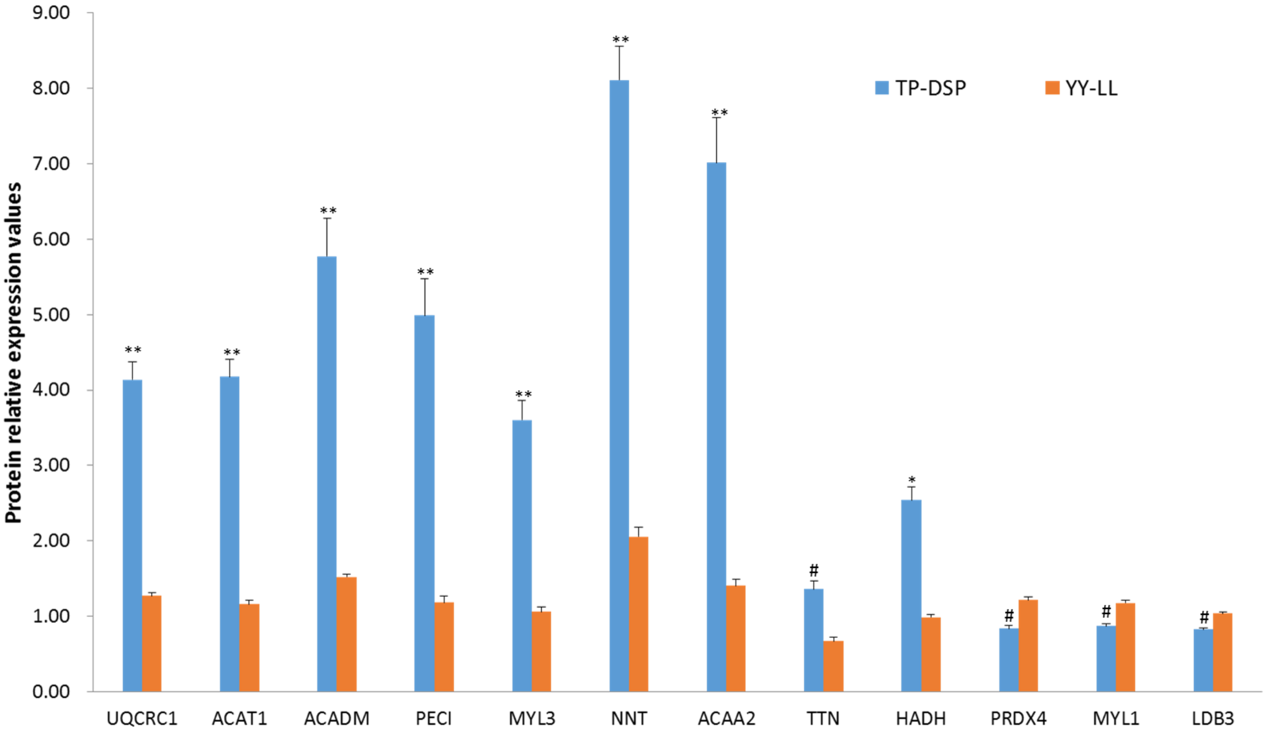

Figure S3. Protein relative expression values in the PRM experiment. ** represents P <0.01, * represents P <0.05, # represents P <0.1 (n=4 animals of each breed).

Supplementary Table S1. Identified peptides in iTRAQ (xml)

Supplementary Table S2. Identified proteins in iTRAQ (xml)

Supplementary Table S3. Differentially expressed proteins between TP-DSP and YY-LL groups (xml)

Supplementary Table S4. Functional annotation of up-regulated DEPs in the TP-DSP group (xml)

Supplementary Table S5. Functional annotation of down-regulated DEPs in the TP-DSP group (xml)
